# When Vitamin Supplementation May Cause More Harm Than Good—An Avoidable Case of Intracranial Hypertension

**DOI:** 10.1002/pdi3.70046

**Published:** 2026-03-26

**Authors:** Kellie Kum Foong Yip, Sameerah Muhammad Samir, Sudipta Roy Chowdhury

**Affiliations:** ^1^ Yong Loo Lin School of Medicine National University of Singapore Singapore; ^2^ The University of Sydney School of Medicine, University of Sydney Sydney Australia; ^3^ Department of Pediatrics KK Women's and Children's Hospital Singapore; ^4^ Duke‐NUS Medical School Singapore; ^5^ Lee Kong Chian School of Medicine Nanyang Technological University Singapore

## 1 Introduction

Idiopathic intracranial hypertension (IIH) is an uncommon cause of headaches in children [1, 2]. Symptoms and signs are a result of increased intracranial pressure (ICP) with otherwise normal cerebrospinal fluid (CSF) composition and absence of evidence of structural abnormalities on neuroimaging [3].

The clinical presentation of IIH in children is variable and sometimes atypical. Pediatric patients can present symptomatically with headache, vomiting, vision loss, diplopia, pulsatile tinnitus, or even asymptomatically, with incidental findings of papilledema [4]. The annual incidence of pediatric IIH is estimated at 0.5–1.2 per 100,000 people per year [5].

The dilemma is that although headaches are a common presentation in the pediatric population, the diagnosis of IIH can be delayed at times due to the need for more invasive procedures, such as lumbar puncture (LP) and additional neuroimaging, which parents and physicians are hesitant to agree to.

In recent times, IIH is diagnosed at increasing rates in all ages, likely due to increasing risk factors of obesity and growing awareness of this condition. Female gender and obesity are well‐established risk factors of IIH in the adult population, but in the pediatric prepubertal population with IIH, these risk factors are not well linked to the condition [6]. The exact disease pathophysiology is still unclear. Research has also shown that vitamin A derivatives, tetracycline‐class antibiotics, recombinant growth hormone, and lithium medications have a strong association with IIH [7].

### 1.1 Case Report

A 10‐year‐old girl presented to our hospital's emergency department with worsening acute‐on‐chronic headaches for 2 months' duration. She had additional symptoms of vomiting for the preceding 2 weeks prior to presentation with decreased visual acuity. Her headache was described as diffuse, pressure‐like, moderately intense, and constant, worsening during periods of physical activity but with no preceding aura. There were no associated seizures, syncope, or neuroregressive symptoms. She had the headaches intermittently throughout the entire day and was unable to sleep due to the pain. Psychosocial history did not reveal any excessive media usage or recent stressful events to contribute to her headaches. She had no infective symptoms, such as fever or chills, to suggest intracranial infections. There was no associated neck tenderness, photophobia, or phonophobia to suggest meningeal irritation. There was no associated family history of migraines. She had a normal developmental history. Because of her increasing pain, she was unable to attend regular school and perform activities of daily living. She was admitted for further investigations and to manage her pain symptoms, as she was not able to function independently due to her headaches.

During her inpatient stay, she continued to have persistent headaches with vomiting and blurring of vision. Her headaches were not relieved with regular analgesia (acetaminophen and ibuprofen). She required regular antiemetics with intravenous hydration due to her poor oral intake with persistent vomiting. There were frequent night awakenings due to her headaches, which further disrupted her sleep.

Neurological examination revealed a cooperative patient who was well oriented with no defects in mentation, speech, or memory. Her gait was normal. Tests of coordination were well performed. Muscle strength was normal and equal bilaterally in all major muscle groups. The reflexes were normal and symmetrical, and there were bilateral plantar flexor responses. Sensation and proprioception were preserved.

The fundoscopic examination revealed bilateral papilledema, but visual field testing was otherwise normal. The pupils were equal and reacted to light briskly. Extraocular movements were full without any nystagmus or ptosis. There was no significant diplopia or sixth nerve palsy on examination.

Blood tests done did not show any significant derangements in her full blood count or electrolyte panel (Table 1). C‐reactive protein was mildly raised, but clinically she remained afebrile. CSF investigations showed normal biochemistry with negative results for bacterial culture and microbiological polymerase chain reaction (PCR) (Table 1).

**TABLE 1:** Summary of the patient's laboratory data.

Variables	Value	Reference range
Blood test		
Hemoglobin (g/dL)	12.9	11.7–14.6
White cell count (10^9^/L)	6.98	5.26–12.25
Platelet count (10^9^/L)	280	140–440
C‐reactive protein (mg/L)	14.2	< 5
Sodium (mmol/L)	137	138–145
Potassium (mmol/L)	4.4	3.4–4.7
Creatinine (μmol/L)	34	27–54
cerebrospinal fluid (CSF) analysis		
Opening pressure (cm H_2_O)	55	6–25
CSF white cell count per μL	0	< 5
CSF protein (g/L)	0.18	0.10–0.40
CSF glucose (mmol/L)	3.6	2.4–4.6
CSF culture	No growth	No growth
Meningitis/encephalitis polymerase chain reaction (PCR)	Negative	Negative

**FIGURE 1:**
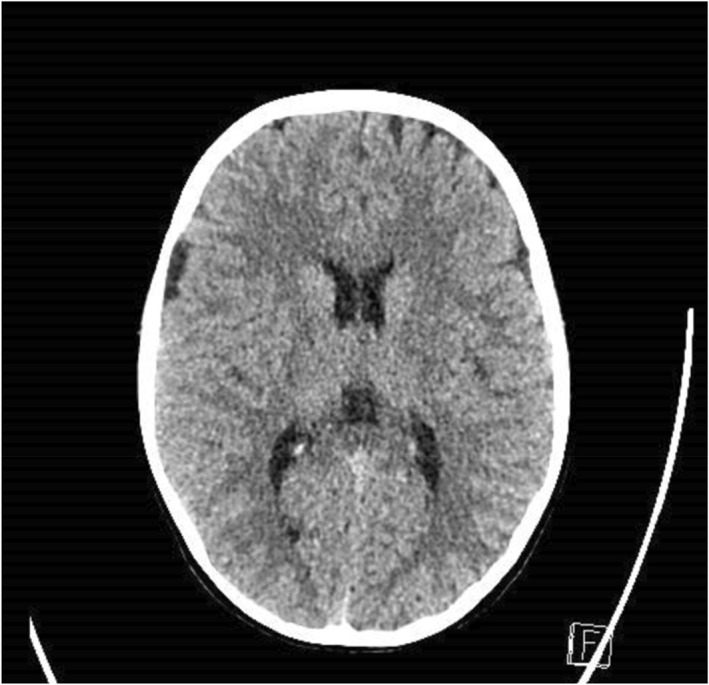
CT brain scan did not reveal any intracranial lesions or bleeds. CT, computed tomography.

**FIGURE 2:**
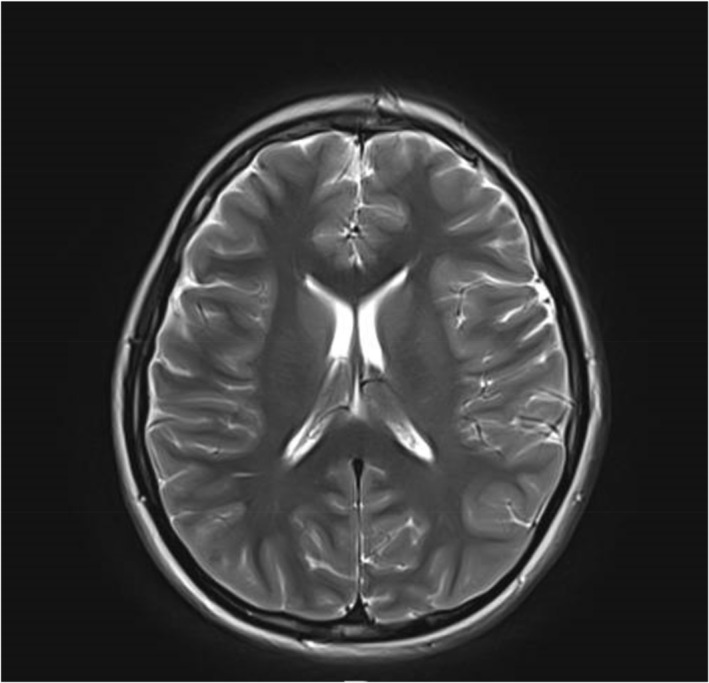
MRI brain did not reveal any significant abnormality. MRI, magnetic resonance imaging.

A computed tomography (CT) scan of the brain was performed to look for any possible intracranial pathology (Figure 1). Magnetic resonance imaging (MRI) of the brain with venography was done with no evidence of venous sinus thrombosis or other structural etiologies (Figure 2). A LP was eventually performed, as there were concerns of IIH raised due to the bilateral papilledema with no discernible abnormality on neuroimaging.

Her CSF opening pressure was 55 cm H_2_O. CSF investigations, including biochemistry and microbiology, were unremarkable with no evidence of intracranial infections (Table 1).

A further detailed history revealed that she had been taking multivitamin gummies, which included vitamin A, for almost 4 years for her general health and well‐being, as she was a fussy eater and underweight. She took 900 mcg of vitamin A daily from her gummies alone, which exceeded the recommended dietary allowance (RDA) (Table 2). This is on top of a diet that was already rich in vitamin A, as her parents were giving her multiple servings of dairy products and cod liver oil frequently. Regrettably, serum or CSF vitamin A level was not obtained.

**TABLE 2:** Recommended dietary allowance for vitamin A in children.

Age	Vitamin A supplementation (mcg)
6–12 months	350
1–6 years	400
7–17 years	600
Acute toxic ingestion	> 20 times the RDA for children

We considered other causes of intracranial hypertension (IH) such as anemia, obstructive sleep apnea, and endocrine disorders (hypothyroidism, hypoparathyroidism, Cushing's disease, and Addison's disease), but her clinical history and blood investigations were not suggestive of these possible differentials.

In view of the above nutritional information, it was most plausible that her headaches arose from IIH secondary to chronic vitamin A supplementation. She was advised to stop her additional vitamin A supplementation and was advised on an appropriate well‐balanced diet for her growth concerns. In view of her progressive symptoms with IH, she was started on oral acetazolamide to prevent any subsequent visual loss. She was discharged after a week of hospitalization when her vomiting resolved with improving headaches. Subsequently, she was given regular follow‐up to monitor her symptoms on an outpatient basis. She required acetazolamide for 4 months until her headaches completely resolved, together with resolution of her papilledema.

## 2 Discussion

The first case of IIH induced by vitamin A was described in 1856 by Elisha Kane, an Arctic medical explorer who reported vertigo and headache after consuming polar bear liver [8].

Vitamin A can be found in many foods, such as liver, dairy products, egg yolks, fish, and green vegetables [9]. Vitamin A derivatives have been used for the treatment of acne, common cold prophylaxis, treatment strategies during measles infection, and as general supplementation in picky eaters or malnourished patients. The RDA of vitamin A for children (1–17 years) ranges from 400 to 600 μg [9]. Our patient had taken a diet rich in vitamin A with almost 900 μg of vitamin A supplementation daily for the preceding 4 years, which could have resulted in excessive levels of vitamin A in her body.

Although the precise pathogenesis of IIH remains unclear, etiologies include elevated intracranial venous pressure, increased CSF outflow resistance, systemic factors such as sleep apnea, and endocrine abnormalities such as excessive corticosteroids or vitamin A. A systematic review in 2020 implicated vitamin A derivatives among a group of drugs strongly associated with drug‐induced IIH [7]. For decades, a link between vitamin A overdose and raised ICP has been explored. Moreover, some of the side effects of treatment with isotretinoin (vitamin A analog) can resemble the classic symptoms of raised ICP [10].

Vitamin A metabolism is a complex process and involves multiple enzymes, binding proteins, and receptors. Vitamin A derivatives are postulated to result in raised ICP through increasing CSF production within the choroid plexus, as well as decreasing its uptake. Retinol is a major form of vitamin A present in humans and other mammals. Retinol is metabolized into retinaldehyde and then to all‐trans‐retinoic acid (ATRA) [11]. The meninges and choroid plexus are the primary sites of ATRA production in humans.

ATRA plays a critical role in regulating gene expression of more than 500 genes upon its binding to specific nuclear receptors. ATRA is thought to increase the expression of aquaporin‐1 in the choroid plexus, thus increasing secretion of CSF into the subarachnoid space [12]. It is also thought to increase gene expression of a molecule in arachnoid granulations within the dural sinuses, which increases resistance to absorption of CSF [9]. Thus, the overexpression of ATRA is postulated to cause IH through various pathways of gene expression.

Our patient in this case study did not satisfy the typical patient phenotype for IIH of an overweight woman of childbearing age. She was a prepubertal child with a low BMI (< 3% for age), which had worried her parents to start her on excessive multivitamin supplementation with a diet rich in dairy and eggs with cod liver oil, which are known to be rich in vitamin A. Thorough workup also alluded to no other underlying medical conditions or prescription medications that could result in the development of IIH. The only plausible cause was her specific diet with excessive supplementation of vitamin A that could have contributed to her symptoms of IIH with worsening headaches.

We regretfully did not manage to obtain blood or CSF levels of vitamin A in our patient. However, much literature has also shown that vitamin A levels measured via serum retinol level have been known to be an unreliable marker, as there are case reports of hypervitaminosis A with normal serum levels [13, 14]. Our review of the literature found conflicting results among previous studies correlating vitamin A or retinol levels in serum or CSF in IIH patients [11].

Thankfully, the literature suggests that most patients with vitamin A toxicity would make a recovery within 1–4 months of cessation of vitamin A supplementation with complete resolution of symptoms [13, 15].

## 3 Conclusion

IIH is an uncommon yet important cause of headaches in children. When identified, timely treatment should be instituted with the main goals of alleviation of symptoms and preservation of vision. Given the strong association between vitamin A supplementation and IIH, as with our patient above, it is crucial for clinicians to be vigilant in inquiring about the use of topical preparations of vitamin A or oral vitamin supplementation in patients suspected of having IIH. Additionally, it is important for clinicians to be aware of this association and to forewarn patients against the use of excessive vitamin A supplementation.

## Author Contributions

S.R.C. managed the case, collected clinical data, and prepared the initial manuscript draft. S.M.S. and K.K.F.Y. assisted with literature review and manuscript editing. S.R.C. supervised the study and provided critical revisions to the manuscript. All authors approved the final version of the manuscript.

## Funding

The authors have nothing to report.

## Ethics Statement

The authors have nothing to report.

## Consent

Written and verbal parental consent was obtained for sharing of the patient's data and for publication.

## Conflicts of Interest

The authors declare no conflicts of interest.

## Data Availability

The datasets used and/or analyzed during this study are available from the corresponding author upon reasonable request.
